# Carbon Sequestration Enhancement by Irrigation in a Mediterranean Pine Forest

**DOI:** 10.3390/plants15050722

**Published:** 2026-02-27

**Authors:** Rafat Qubaja, Murray Moinester

**Affiliations:** 1 Department of Plant & Environmental Sciences, Weizmann Institute of Science, Rehovot 76100, Israel; 2 School of Sustainable Engineering and the Built Environment, Arizona State University, Tempe, AZ 85287, USA; 3 School of Sustainability, Arizona State University, Tempe, AZ 85281, USA; 4 Center for Hydrologic Innovations, Arizona State University, Tempe, AZ 85287, USA; 5 School of Physics and Astronomy, Tel Aviv University, Tel Aviv 69978, Israel; murray.moinester@gmail.com

**Keywords:** drylands forest, carbon sequestration, organic carbon, inorganic carbon, irrigation

## Abstract

Sequestration of atmospheric CO_2_ in the Yatir Mediterranean semi-arid Aleppo Pine Forest (*Pinus halepensis*) close to the border of the semi-arid timberline was characterized and quantified under field conditions. Measurements of organic and inorganic CO_2_ sequestration with gas exchange, stock counting approach, and remote sensing were made in both rainfed control (~12% average annual Soil Moisture) and long-term experiment of irrigation (~10 years with ~24% annual average SM) plots, providing the opportunity to separate the effects of atmospheric water demand from soil water stress on the atmospheric CO_2_ sequestration responses. Measurements yield an organic carbon sequestration (OCS) rate of ~550 g CO_2_ m^−2^ yr^−1^, 60% in soil and 40% in biomass (standing and removed). In addition, measurements yield an inorganic carbon sequestration (ICS) rate of ~65 g CO_2_ m^−2^ yr^−1^ (for half meter soil depth) via calcite (CaCO_3_) precipitation in the soil due to root exhalation of CO_2_ (25%) and microbial activity (75%). The drip-irrigated plot showed approximately 3 times higher organic CO_2_ sequestration than the control plot, divided equally between the soil and the biomass. For the irrigated plot, the inorganic CO_2_ sequestration rate was ~1.8 times higher than that of the control plot. These measured values demonstrate the relatively high potential for carbon sequestration in Mediterranean drylands forests under irrigated and non-irrigated conditions.

## 1. Introduction

Forestation is a simpler and significantly less expensive method for removing atmospheric CO_2_ than massive and high-tech engineering projects [1]. Drylands are more suitable than temperate lands for forestation efforts to remove atmospheric CO_2_ by organic (OCS) and inorganic carbon sequestration (ICS). Drylands make up over 40% of the global land area [2], covering almost 45 Mkm^2^. They store approximately 30% of all soil organic carbon (SOC) in the world [3,4,5] and approximately 80% of all soil inorganic carbon (SIC) in the world to 2 m depth [5,6,7,8]. The high value for SIC is because calcite (CaCO_3_) washes away in temperate zones but not in drylands. Drylands forests reportedly have great global potential to increase organic carbon stocks under increasing water supply [9]. However, their ability to store large quantities of carbon, how this influences microbial activities, and how the organic and inorganic carbon pools interact are less understood. Drylands would be prime regions for forestation were it not that the climate is hot and harsh and that sufficient water appears to be lacking. Thus, most of these regions have not been previously considered for forestation [10,11].

Climatic conditions in the Mediterranean basin have become drier in recent decades [12], with an up to 20% decrease in total annual rainfall projected by the year 2050 [13]. Such a change would have an important influence on ecological consequences related to both carbon inputs and outputs (e.g., assimilation and respiration of CO_2_ fluxes) [14,15] and could therefore influence the distribution of aboveground and belowground carbon [16]. Irrigation is expanding in drylands [17]. It can expand further if fossil and desalinated water are used [18,19]. However, no studies have previously evaluated how dry-season irrigation affects OCS and ICS in Mediterranean basin forests to the present study’s level. In the few studies conducted on irrigated drylands agricultural fields, significant increases in CO_2_ sequestration were found [15,17,20].

The ecological problems of increasing global warming and ocean acidity are inextricably intertwined with increasing levels of atmospheric CO_2_. Constraining potential atmospheric CO_2_ sequestration estimates is critical to guide future climate change and ocean acidity mitigation actions. CO_2_ is currently being emitted globally at roughly 40 Gt CO_2_ yr^−1^. About 50% of these emissions accumulate in the atmosphere, while the ocean and terrestrial biosphere share the rest about equally [21,22]. The global atmospheric CO_2_ reservoir is presently increasing annually by ~22 Gt CO_2_ [22], primarily due to the release of carbon to the atmosphere from fossil fuel emissions (~90%), from deforestation, and from other land-use-change activities (~10%) [22]. Indeed, the global Conference of the Parties climate pacts call for the world’s nations to significantly reduce CO_2_ emissions to prevent global warming from rising more than 2.0 °C above pre-industrial levels [23]. Achieving this goal also requires actively removing CO_2_ from the atmosphere and storing it long term (sequestration). Therefore, more efforts are needed to enhance the removal and sequestration of atmospheric CO_2_.

The terrestrial biosphere may offer a solution by the sequestration of atmospheric CO_2_ in tree biomass and soils, thereby storing large stocks of global carbon [24,25]. Trees take in atmospheric CO_2_ through the process of photosynthesis, which converts the atmospheric CO_2_ to OC, which comprises almost half of a tree’s dry biomass [25,26]. Organic carbon is transferred to the soil by microbial mediated decomposition of leaf litter, dead wood, tree products, and animal remains to form SOC [27]. In addition, atmospherically derived CO_2_ can be removed and transformed into SIC [20,28]. This occurs when CO_2_ exhaled by the roots or released in organic decomposition processes within the soil column combines with soil moisture to form carbonic acid (H_2_CO_3_). This in turn dissociates rapidly into H^+^ and HCO_3_^−^ bicarbonate [28]. When bicarbonate combines with a calcium to form inorganic calcite (CaCO_3_), some calcite precipitates and is thereby sequestered in the soil column. This occurs when, after evaporation, the activity product of Ca and CO_3_ ions exceeds their solubility product. Another portion of the inorganic carbon, residing as bicarbonate, descends and enters the water table where it is effectively sequestered long term in slow-moving aquifer water. Microbial decomposition also releases and increases the partial pressure of CO_2_ into the soil–gas phase, thereby promoting calcite formation [29,30]. Some microbes use atmospheric CO_2_ to directly precipitate calcite within the desert soil [31].

**Figure 1:**
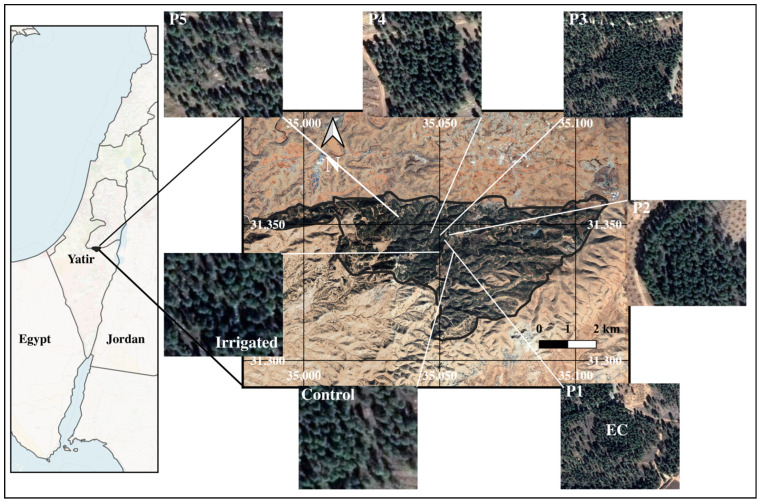
Aerial map of Yatir forest in Southern Israel (~9 km across), indicating the experimental setup, which includes the five sampling plots used to count carbon stock in 2001, 2016, and 2025 studies. The locations of the control rainfed and irrigated plots are also shown, where leaf and soil CO_2_ fluxes were measured since 2014. The eddy-covariance (EC) flux tower was erected in plot 1 in 2000.

The present study is based on a unique, long-term irrigation experiment over a ~10-year period in a mature Mediterranean *Pinus halepensis* drylands forest (since 2016; Figure 1) [23,28]. Our objective is to assess the organic and inorganic carbon sequestration rates in the semi-arid Yatir forest in irrigated (~24% annual average Soil Moisture; high atmospheric water demand) and non-irrigated (~12% average annual SM; high atmospheric water demand) plots. We hypothesize that removing water limitation would accelerate and intensify the carbon cycling of drylands forests. Our results improve understanding of the dependence of the CO_2_ cycle on moisture stress in the soil and atmosphere.

## 2. Results

### 2.1. Soil Moisture and CO_2_ Profile

**Figure 2:**
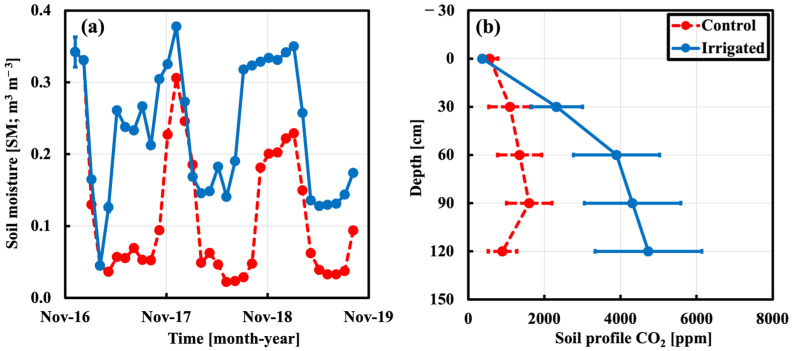
(**a**) Soil moisture for the upper 10 cm of soil. (**b**) Soil CO_2_ profile measured. For control (red dashed) and irrigated plots (blue solid). Error bars represent ±SE of the measurements.

Before starting the irrigation experiment, soil moisture (m^3^ m^−3^) during Year-zero (Year0) showed no significant differences between the two plots. During irrigation, the average SM was maintained higher in the irrigated than in the control plot by a factor of ~2.0 (*p* < 0.001), as shown in Figure 2a. The average ratio of soil CO_2_ (ppm) to depth from 0 to 120 cm depth in the irrigated plot is about 3 times higher than in the control plot (*p* < 0.001), as shown in Figure 2b.

### 2.2. Leaf and Soil CO_2_ Fluxes

**Figure 3:**
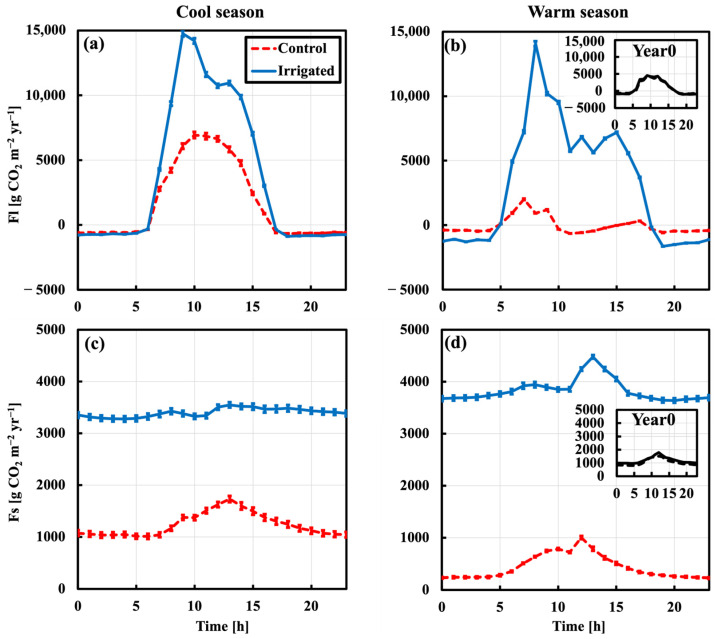
Average seasonal diurnal cycles of (**a**,**b**) leaf (Fl) and (**c**,**d**) soil (Fs) CO_2_ fluxes for control plot (red dashed) and irrigated plot (blue solid) chambers. Error bars represent ±SE of the measurements. The cool season is Oct–Mar (**a**,**c**) and warm season is Apr–Sep (**b**,**d**). The inserts represent the initial differences (average annual diurnal cycles) between the control (black dashed line) and irrigated (black solid line) plots, in b for Fl and in d for Fs, before beginning irrigation (Year0).

Figure 3 shows the average seasonal diurnal cycles of leaf flux integrated CO_2_ rate (Fl) and soil CO_2_ flux (Fs) for control plot (dashed) and irrigated plot (solid) chambers. Before starting the irrigation experiment, the initial values of FI and Fs (Year0) showed no significant differences between the two plots (Figure 3 inserts). After beginning irrigation, the annual average Fl was measured as 3340 g CO_2_ m^−2^ yr^−1^ and 788 g CO_2_ m^−2^ yr^−1^ in the irrigated and control plots, respectively (a factor of 3.3, *p* < 0.001). Daytime uptake (positive values) was higher than nighttime release (negative values) by factors of 8 and 4 in the irrigated and control plots, respectively (Figure 3a,b). The average annual Fs was 3616 g CO_2_ m^−2^ yr^−1^ and 833 g CO_2_ m^−2^ yr^−1^ in the irrigated and control plots (Figure 3c,d), respectively (a factor of 4.1, *p* < 0.001). Enhancement values depend on the season, with higher differences in the warm season than the cool season for Fl and Fs (Figure 3a–d).

**Figure 4:**
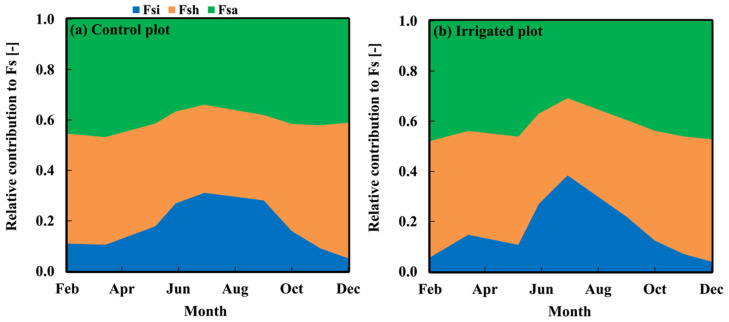
Seasonal variations in the relative contribution of soil autotrophic (Fsa), heterotrophic (Fsh), and abiotic (Fsi) components to Fs in (**a**) control plot and (**b**) irrigated plot.

Figure 4 shows the seasonal variations in Fs partitioning of CO_2_ emissions between autotrophic (Fsa), heterotrophic (Fsh), and abiotic (Fsi) components. The annual averages of relative contribution of Fsa, Fsh, and Fsi were 0.41, 0.42, 0.17, respectively, without significant differences between the control and irrigated plots. The annual averages of Fsa, Fsh, and Fsi fluxes in the irrigated plot were 2.9, 2.7, and 2.5 times the control plot values, respectively.

### 2.3. Organic Carbon Stock and Flux

Table 1 shows the results based on the organic carbon stock (OCS) measurements. The EC and OCS measurements agreed to within 9%. These data were collected over a 10-year period. The forest accumulated about 550 g CO_2_ m^−2^ yr^−1^ over the study period in the control plots (Table 1), with 60% and 40% sequestered in soil and biomass (standing and removed), respectively. In the irrigated plot, organic carbon was sequestered in soil and biomass (standing and removed) at equal rates. Both aboveground and belowground CO_2_ inputs increased under irrigation. Significantly, the irrigated plot shows an ~3 times higher annual organic CO_2_ sequestration rate compared to the control plot (Table 1).

### 2.4. Inorganic Carbon Sequestration and Soil Profile

**Figure 5:**
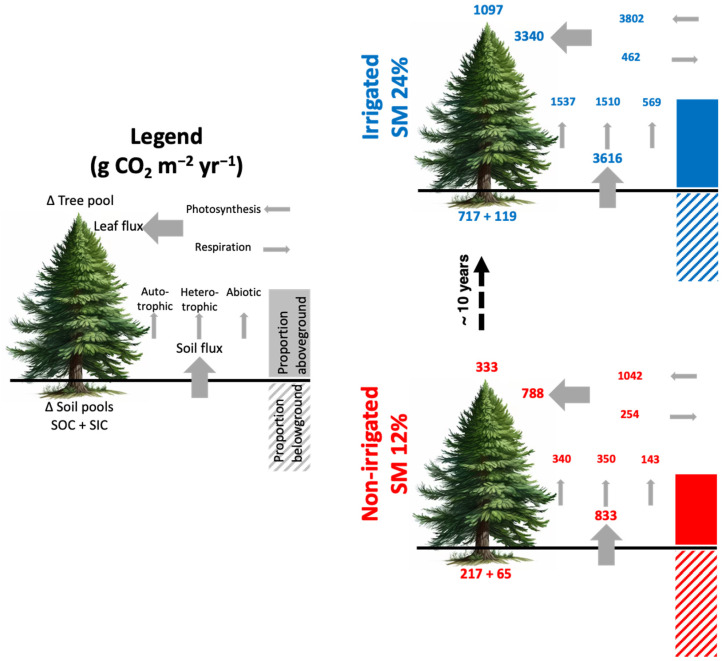
Relative distribution of CO_2_ (g CO_2_ m^−2^ yr^−1^) for control plot (red) and irrigated plot (blue) measurements in different pools and fluxes, as well as the overall above and belowground distribution, are shown in non-irrigated and irrigated plots. Total ecosystem CO_2_ sequestration (TCS) is the sum of tree and soil pools, which equals to the sum of organic and inorganic carbon.

For the forest’s inorganic carbon sequestration, the calcite deposition rate into the sediment yields a sequestration rate of ~15 g CO_2_ m^−2^ yr^−1^ for a half-meter depth of root exhalation in Yatir. In addition, we estimate that ~50 g CO_2_ m^−2^ yr^−1^ could potentially be precipitated in the soil as calcite as a result of microbial activity to 0.5 m depth. In total, the inorganic carbon sequestration rate is then ~65 g CO_2_ m^−2^ yr^−1^ in the rainfed plot; while in the irrigated plot, it is higher than the control plots by a factor of ~1.8 (Figure 5).

### 2.5. Total Atmospheric Carbon Dioxide Sequestration

**Figure 6:**
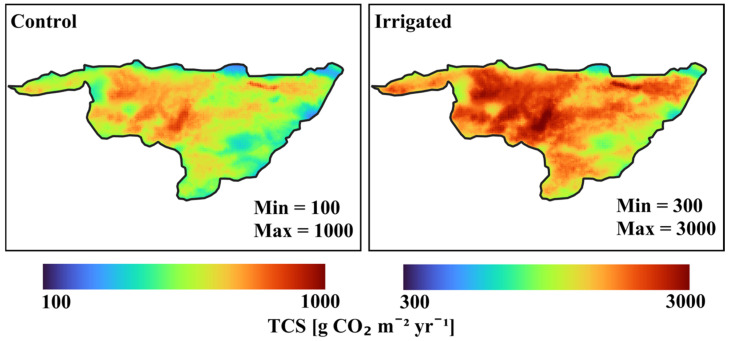
Spatial variability of the total carbon sequestration (TCS) = OCS + ICS for control plot (**left side**) and irrigated plot (**right side**) measurements.

Figure 5 shows the comparisons of total ecosystem CO_2_ sequestration (TCS) of non-irrigated rainfed and irrigated plots. All values shown are in units g CO_2_ m^−2^ yr^−1^. Total carbon sequestration (TCS) from rainfed and irrigated plots are 615 (217 + 65 + 333) and 1933 (717 + 119 + 1097) g C m^−2^ yr^−1^, respectively. Soil flux irrigated is 3616 (1537 + 1510 + 569) and soil flux non-irrigated is 833 (340 + 350 + 143); meanwhile, irrigated leaf flux is 3340 (3802 − 462) and non-irrigated is 788 (1042 − 254). The uncertainties in all these values are ~15%. We used data of remote sensing and applied their spatial variability to the TCS of the control and the irrigated plots in order to determine the spatial variability of the carbon sequestration. We found that the SD of spatial variability for control and irrigated plots were 13% from TCS (Figure 6). Figure 6 shows large differences between different areas within Yatir and a factor of ~3 higher TCS rate due to irrigation.

## 3. Discussion

The present study took advantage of a unique, long-term irrigation experiment in a mature Mediterranean (Yatir) *Pinus halepensis* dryland forest. This study focuses on total carbon sequestration (TCS) components. We evaluate the potential for atmospheric CO_2_ removal by both organic (OCS) and inorganic (ICS) sequestration processes on irrigated and non-irrigated plots.

### 3.1. Leaf and Soil Fluxes

The leaf fluxes (Fl) show a strong diurnal cycle with daytime carbon sink and nighttime carbon source in both plots (Figure 3a,b). The Fl enables the examination of instantaneous response of plants between the control vs. irrigated plots to environmental conditions, such as excessive vapor pressure deficit (VPD) under both water scenarios. Results showed that the high decrease in Fl from cool to warm season was followed by the decrease in soil moisture, with progressive increase in VPD for the control plot, while the low decrease in the irrigated plot was followed by the progressive increase in VPD without water stress. Furthermore, the phenomenon of “midday depression” was caused by high VPD that was often linked to limited water supply [32,33], which led to the closure of stomata to conserve water at the expense of reduced carbon uptake. This phenomenon was not strong with lower atmospheric water stresses without water-limited supply in wet season for both plots (Figure 3a). During the dry season, unlike the irrigated plot, the control plot showed the midday depression phenomenon (Figure 2b), which indicated the distinct reduction in CO_2_ uptake at midday. The midday depression was discussed by previous studies based on in situ observations and found for different ecosystems and across biomes [32,34].

Similarly, the soil fluxes (Fs) showed a strong diurnal cycle with a continuous source of carbon from both plots (Figure 3c,d). The large decrease in Fs from cool to warm season was followed by the decrease in soil moisture for the control plot, while there was a minor change in the irrigated plot without water stress, which was also observed by [35,36,37,38]. Unlike Fl and Fs, the relative contribution of soil components did not show significant changes between plots in Figure 4, while their fluxes were increased significantly as found by [15,35,36,37,38].

These findings, leaf with soil and its sources fluxes, supported with the long-term studies, were conducted in a dry climatic region on a pine forest with ~twice the precipitation rate and standing tree concentration compared to the Yatir site [35,36,37,38]. They found that the decade-long irrigation in this dry forest doubled Fl and Fs. Normalizing for the standing tree density (~2.4), the difference of these values between our study’s irrigated and control plots would be increased by a factor of ~5. Also, irrigation resulted in a three- to four-fold increase in the amount of CO_2_ assimilates transferred to and respired from the soil—suggesting that irrigation treatment is primarily driven by increased autotrophic and heterotrophic respiration [35]. Relatedly, soil components could respire 10 to 13 times more CO_2_ in irrigated soils compared to the dry control [15]. Furthermore, Qubaja (in progress) studied the effects of reducing the amount of precipitation on five major Mediterranean tree species in Yishi Forest. Their preliminary results show that the Fl and Fs fluxes drop by half with reduced water supply. Overall, these positive irrigation effects imply that water supply for the dryland forest enhances atmospheric CO_2_ sequestration while the naturally dry conditions in this drylands forest strongly suppress the carbon cycling rates.

### 3.2. Irrigation Alters the Organic Carbon Sequestration

Despite over 65 years of soil water deficit and high atmospheric water demand, significant organic carbon sequestration (OCS) rates were observed in long-term irrigated and non-irrigated plots. The mean annual OCS rate based on stock-based measurements represents the average increase in carbon in the soil and biomass (standing and removed) over the ten-year observation period. Long-term carbon stock is ~40% in control and 60% in irrigated plots, against the global forests’ C stock density (globally ~22.8 kg C m^−2^) and lower than the range of other forests (Boreal, Temperate, and Tropical forests) [39]. With considering the stock changes in both plots, this needs 90 and 20 years for control and irrigated plots, respectively, to reach global forests’ C stock density level [39,40]. Dryland soils are ideal candidates for atmospheric CO_2_ sequestration because unlike temperate systems, they are likely much below their SOC storage capacity compared to wetter climates [39,41]. Relatedly, long-term studies in drylands found that irrigation increases SOC by a factor of 3–5 [25,30,35,37,38,41,42]. They found that the decade-long irrigation increased litterfall, fine-root production, and belowground carbon allocation with a factor between 3 and 5. In this regard, previous drylands afforestation models merit reconsideration. The organic carbon residence time for this sequestration is more than 100 years, considering the typical long life-span of pines and their decomposition time after falling. Thus, the Yatir Mediterranean pine forest is effective at sequestering carbon, both as total standing biomass as well as in the soil [43,44,45,46].

### 3.3. Irrigation Enhances Inorganic Carbon Sequestration

In the present study, two sources of inorganic carbon were studied and quantified. For the root exhalation process, inorganic carbon was precipitated as calcite in the soil due to the formation of soil carbonic acid that arises from the reaction of soil water with CO_2_ exhaled from tree roots, which originally was sequestered from atmospheric CO_2_ [47]. Tree roots extend downwards to much greater depths in drylands compared to temperate zones [48]. From a study in hyper-arid conditions on mature acacia trees (*Acacia tortilis* and *Acacia raddiana*) growing in the Arava desert in Israel, with water isotopes (δ^18^O, δ^17^O and δ^2^H), the vertical root system was estimated to reach around 8.0 m below the soil surface (Qubaja, in progress), and 6 m global average depth of root respiration in drylands [37,38]. In drylands, ref. [49] noted that the downward CO_2_ fluxes are strong. Further, the increased depth of the root systems in drylands facilitates more rapid weathering of the underlying lithology and the minerals within the soils [48,50]. In humid regions, where rainfall is plentiful, this precipitate dissolves, while in drylands regions, where rainfall is sparse and irrigation is modest, precipitated calcite can remain stable for the long term [51].

Soil microbes have long been known to precipitate calcite [52]. The processes by which soil microorganisms utilize atmospheric CO_2_ to precipitate calcite have been thoroughly reported [53,54]. Sequestration of atmospheric CO_2_ by soil microorganisms can be long term and inexpensive, with significant rates, under laboratory and field conditions, as described in recent studies [31,55]. The major factors inhibiting soil microbial activity are the intense irradiation and lack of moisture and organic carbon in drylands [56], which could be improved by planting tress [57,58,59]. These microbes play a vital role in breaking down organic matter and precipitating calcite within the soil column [28]. Unfortunately, representative soil profiles in drylands forests showing the concentration of calcite-precipitating microbes are not available. It is possible that, over time, the microbial communities will increase in mass and descend through the soil profile to greater depth. Thereby, the microbial contribution would increase proportionally.

Our results of total inorganic CO_2_ sequestration, in line with many studies, have shown that inorganic carbon sequestration takes place in drylands regions, which have sparse vegetation and poor soil. The measured rate is around 370 g·CO_2_ m^−2^ yr^−1^ in USA and Chinese deserts [28,60]. Ref. [35] found in a decade-long irrigation study conducted in a dry pine forest that the inorganic carbon sequestration rate increased by a factor of 1.5 compared to the control plot. In another study, ref. [61] found that after 40 years, in irrigated soil, the calcite concentrations were high in soil cores. A long-term study in arid-calcic cropland found that flooding irrigation increases inorganic soil carbon about 4 times compared to unirrigated fields [41]. Ref. [30] found an increase of SIC by 40–60% over 30 years in irrigated arid and semi-arid crop fields. Many other studies inferred that irrigated dryland has a considerable inorganic carbon sequestration potential, due in part to leaching and deposition in the lower part of the soil [29].

### 3.4. Uncertainty of Carbon Sequestration

The relatively small difference (~9%) between the two independent approaches, OCS based on stock-based approach vs. EC tower, and the long-term perspective indicate small uncertainty and provide confidence in these widely used methodologies. This difference was within the uncertainties estimated for the two approaches and consistent with previously reported uncertainties [27]. Furthermore, the small difference (~13%) between the stock-based vs. remote sensing approaches showed clear spatiotemporal overlap of the TCS. The compatible results of the three methods suggests that the level of uncertainty is less than 20% [27,62].

### 3.5. Irrigation Limitation and Implications in Drylands

The utilization of the essentially open spaces of drylands for potential widescale afforestation has been deemed unfeasible due to the apparent lack of water and was restricted to <10% of the global drylands [10,11]. While expressions of surface water are indeed lacking, as is appreciable rainfall, this conclusion fails to take into consideration the large resources of groundwater contained in aquifers that immediately underlay these regions. These aquifers are not related to the present meteoric regimes. Such waters underly drylands regions globally [63,64], e.g., Nubian Aquifer System residing under the Sahara Desert [65]. It is suffice to say that sufficient useable groundwater is available for afforestation of drylands [66], which has until now been removed from consideration.

Using such fossil water for forest irrigation would enhance the cooling effect benefit of carbon sequestration and would partially offset the surface albedo effect while enhancing the water cycle [10,67,68]. For example, the evapotranspiration may stimulate dense low-lying clouds formation, which, being strongly coupled to the ground, would reflect solar radiation [69]. Irrigation in turn may eventually spur increased precipitation. Taking such factors into account, ref. [70] claims that previous authors overestimated the climatic impact of the albedo effect. In this regard, previous afforestation models about the area of total drylands available for afforestation merit reconsideration [10,71].

## 4. Methods

### 4.1. Study Site

Yatir forest is a 28 km^2^ Aleppo pine forest growing successfully at the semi-arid timberline with no irrigation or fertilization, shown in Figure 1 [72]. Jewish National Fund (Keren Kayemeth LeIsrael) foresters have planted ~4 million trees at Yatir since 1965 [72]. This site (GPS: 31.34 N, 35.05 E) is situated above a carbonate mountain aquifer at an elevation of ~650 m, at the edge of the Negev desert. It is the largest forest in Israel [72].

In 2016, the tree density was ~30,000 trees km^−2^ (1 tree per 6 × 6 m^2^); tree height and diameter at breast height were ~9.0 ± 1.5 m and ~19.7 ± 5.0 cm (Mean ± SD), respectively; leaf area index (LAI) was ~1.5 ± 0.3 [27]. The soil covering an underlying chalk and limestone base has a thickness that generally ranges from 0.2 m to 1.0 m, though locally thicker soil profiles up to 4.5 m were also encountered. The soil itself has a clay-loam texture (41% silt, 31% sand, and 28% clay) with a bulk density of ~1.65 g cm^−3^. The soil has a high stoniness fraction of over 45%, with an abundant limestone content. Limestone outcrops occur over approximately a third of the area [27]. The mean annual precipitation (P) is ~285 mm, falling solely during the cool season (October to March) as high-intensity events, while the warm season (April to September) is hot and dry. The mean annual potential evapotranspiration (PET) is 1600 mm [73,74]. Conditions are at the drier and hotter limit (Aridity Index AI = P/PET = 0.18) compared to the world’s drylands (AI ≤ 0.5).

### 4.2. Irrigation Experiment

Since 2016, a 30 m × 35 m plot adjacent to an eddy flux covariance site was irrigated to maintain an average annual Soil Moisture (SM) volumetric water content of ~24% at 10 cm depth, with water supplied continuously via drip irrigation. A site 30 m away from the irrigated plot and similar in size, slope, and composition was established as a rainfed control. The control plot typically experiences an average annual ~12% SM at 10 cm depth. Both irrigated and control plots contain 30 Aleppo pine trees.

In 2014, before irrigation, we measured SMs and leaf and soil fluxes in these two plots to determine the baseline differences between the two plots. These measurements are referred to as Year-zero (Year0).

### 4.3. Leaf Chamber Measurements

From 2014, integrated leaf flux (Fl) measurements of CO_2_ were carried out in the control and irrigated plots to capture both photosynthesis and respiration processes over chosen time periods in a sealed environment around the leaves. Identical branch chambers were positioned at the top of the canopy in both plots. New pine tree branches were placed in these chambers every month. The branches were held by plastic netting to flatten the branch structure and expose leaves (needles) to roughly equal light and humidity conditions. The chambers were opened and closed on an automated hourly schedule. Every hour, the chambers were closed for 4–8 min. Measurements were taken in the closed state and in the open state before and after the closed state. Air within the chambers in the closed state was mixed by two fans, and the chambers were sealed with Viton (synthetic rubber) O-rings. Ambient air entered the chambers through small holes along the wall opposite the exit. Chamber effluent was analyzed using a quantum cascade laser (QCL, Aerodyne Inc., Billerica, MA, USA) capable of quantifying CO_2_ mole fractions [75]. Leaf area was determined by measuring the mean length and diameter of the leaves for each new branch. More system specifications are given in [33].

### 4.4. Soil Chamber Measurements and Partitioning

From 2014, soil CO_2_ fluxes (Fs) were measured in the control and irrigated plots with automated systems using opaque chambers and a multiplexer (LI-8150, LI-COR, Lincoln, NE, USA). The chambers were closed for 2 min on PVC plastic collars of 20 cm diameter that were preinstalled airtight into the soil over the entire plot. They were otherwise positioned away from the collars. Soil fluxes in the plots were measured by means of three chambers that were rotated between 21 collars. Data were recorded on a half-hour basis (48 times daily). Air from the chambers was circulated through an infrared gas analyzer to record CO_2_. More specifications about calibration and upscaling of the 21 collar measurements (under trees, between trees, open areas) to overall plot-scale soil CO_2_ flux are given in [73].

A linear mixing model was employed to estimate autotrophic (Fsa; carbon loss during growth and non-growth-related respiration by plants), heterotrophic (Fsh; decomposition of litter and soil organic matter), and abiotic (Fsi; CO_2_ released from non-biological processes, such as chemical reactions in the soil) contributions to the total Fs flux from soil to atmosphere [73]. The model used δ^13^C to identify different carbon sources, Δ^14^C to determine the age of carbon, and incubation (cultivated) data collected monthly at controlled soil conditions. The model is based on Equations (1)–(3), where f indicates the fraction of total soil CO_2_ flux (e.g., *f*_sh_ = Fsh/Fs), while subscripts sa, sh, and si indicate autotrophic, heterotrophic, and abiotic components, respectively.

(1) $$\delta^{13}C_{Fs}=f_{sa}*\delta^{13}C_{sa}+f_{sh}*\delta^{13}C_{sh}+f_{si}*\delta^{13}C_{si}$$

(2) $$\Delta^{14}C_{Fs}=f_{sa}*\Delta^{14}C_{sa}+f_{sh}*\Delta^{14}C_{sh}+f_{si}*\Delta^{14}C_{si}$$

(3) $$1=f_{sa}+f_{sh}+f_{si}$$

This set of three equations was used to solve for the three *f* fractions.

### 4.5. Organic Carbon Stock Measurements

The original Yatir forest organic carbon sequestration (OCS) assessments were performed in 2016 [27,76] on the same five 30 × 35 m plots in the central part of the forest (Figure 1). Detailed descriptions of these assessments were given previously [27], including estimates of standing biomass, litter, soil, and removal components.

The mean annual OCS rate is calculated as the sum of the differences in soil, tree biomass CS, and the carbon removed (grazing, mortality, thinning, and sanitation) and then divided by the observation period (~10 years).

Standing biomass: Organic carbon sequestration by trees was estimated by counting the number of trees, measuring their diameters at breast height (DBH), measuring the heights of trees in each plot, and then applying site-specific allometric equations [27,76]. The dependent variable in these equations was biomass, and the main independent variables were tree DBH and tree height. Figure 1 illustrates the plots of interest for this method. The understory contribution to the forest CO_2_ sequestration typically includes shrubs and small trees, young trees, and herbaceous plants. Its contribution to carbon sequestration was previously estimated to be negligible [27,76].

Removal biomass: Carbon removal due to the thinning, mortality, and sanitation was estimated based on local forestry management records available for 2001 to 2025 [72]. Carbon removal components were counted in the same manner as in the previous studies conducted on the same plots [27].

Soil: The surface litter layer was sampled within a grid of 40 × 40 cm, taking 7 samples from each plot, and the litter was counted as part of soil organic carbon (SOC). To estimate SOC, seven cores (0.5 m depth) were collected from each plot [27]. Concentrations of total soil C was determined in an elemental analyzer (EA 1108, Carlo-Erba, Milan, Italy). SOC was measured by the EA 1108 following removal of carbonates with 1 N HCl for 24 h according to [27,76]. For more details about the soil sample processing protocol and its counting for CO_2_ sequestration, see [27].

### 4.6. Inorganic Carbon Measurements

Inorganic C concentration was determined as the difference between total soil C and SOC in Section 4.5. The inorganic CO_2_ sequestration rate (ICS) via calcite precipitation (CaCO_3_) in the soil was determined by measuring the decrease in the dissolved bicarbonate concentration as a function of depth as soil water percolates downward [47,77,78]. Soil water samples were collected in depth profiles extending from the surface to a maximal depth of ~4.5 m. Carbon isotope ratios δ^13^C and Δ^14^C were measured as a function of depth in the liquid and solid phases of soil profiles in the unsaturated zone. The techniques developed and used for sampling the soil moisture and the inorganic carbon via isotopic measurements are presented in [78]. The depth profiles were converted to time profiles, using the measured percolation rate, combined with the data from the mid-core depth (2.2 m) as the representative [77].

### 4.7. Eddy-Covariance Approach

An eddy-covariance (EC) flux tower at the center of the forest was erected in 2000 following Euroflux methodology [27,73,79]. The EC method is a key atmospheric measurement technique employed to determine net vertical forest–atmosphere exchange fluxes of CO_2_, water vapor, heat, etc. The system used a 3D sonic anemometer to measure wind velocity (Omnidirectional R3, Gill Instruments, Lymington, UK) and a model 7000 CO_2_ infrared gas analyzer (LI-COR Inc., Lincoln, NE, USA) to measure daytime and nighttime net ecosystem CO_2_ exchange (NEE). The EC tower’s footprint analysis indicates that 95% of the flux originates within the forest boundaries [27]. The NEE measurements were used to estimate the annual net ecosystem production (NEP). The annual CO_2_ sequestration was obtained by integrating half-hour values after U* night-time correction and quality control [73]. The U* correction is a threshold value of friction velocity that defines the minimum wind speed necessary for reliable EC measurements.

### 4.8. Total Carbon Sequestration

We used the organic carbon sequestration (OCS) from Section 4.5 to validate the flux tower NEP from Section 4.7. Total atmospheric CO_2_ sequestration (TCS) was constructed based on two sets of terms: (i) the sum of OCS from Section 4.5 and ICS from Section 4.6, and (ii) the sum of the soil and biomass (standing and removed) pools.

Using the Moderate Resolution Imaging Spectroradiometer (MODIS) data, and assuming organic and inorganic carbon sequestrations are spatially correlated, we combined them to create maps of TCS based on the control and irrigated plots scenarios. Long-term remote sensing studies conducted at Yatir are described in [80].

### 4.9. Statistical Analyses

The statistical analyses were carried out using MATLAB software, Version R2023b (MathWorks, Inc., Natick, MA, USA). Satellite images were analyzed with Panoply NASA (version 5.2.2 released in 2022) and QGIS 2022 (Open Source Geospatial Foundation). Statistical indices of linear regressions of irrigated plot variables against control plot variables are the slopes (*b*, unitless); while *p* defines the significance level.

## 5. Conclusions

This study quantified the long-term potential and significant organic and inorganic CO_2_ sequestration rates of a Mediterranean pine forest at the semi-arid timberline. Reducing dry-season soil moisture stress, with summer supplemental irrigation, was shown to correlate well with increasing CO_2_ sequestration rates and reshaped carbon cycling within the plant–soil system. This long-term study showed a continuing CO_2_ sequestration efficiency 65 years after planting the forest. These results support the potential for sustainable forest productivity in degraded drylands zones, areas that are also expected to expand and undergo significant drying trends in the future. It will be an important direction for future carbon research to fully understand the drylands carbon sequestration mechanism at ecosystem, regional, and global scales. We emphasize the need for further drylands forest sequestration measurements and the need to begin implementing a global land management policy of widespread forestation in drylands regions.

## Figures and Tables

**Table 1:** Breakdown of organic carbon stock measurements (g CO_2_ m^−2^) based on Section 4.5 in 2025. Stock change (∆) refers to the 2016 study (g CO_2_ m^−2^ yr^−1^) averaged over ~10-year period.

	Organic Carbon Stock (OCS)					
	Control			Irrigated		
Compartment	OCS	%	Δ	OCS	%	Δ
Trees + Cleaning	14,368	~40	333	22,119	~50	1097
Soil	20,082	~60	217	25,143	~50	717
Sum of OCS	34,450	100	550	47,263	100	1814

## Data Availability

All data are available in the text and cited references.
